# Comparison of myocardial tagging and feature tracking in patients with severe aortic stenosis

**DOI:** 10.1186/1532-429X-15-S1-P100

**Published:** 2013-01-30

**Authors:** Christopher Schneeweis, Tomas Lapinskas, Bernhard Schnackenburg, Alexander Berger, Thomas Hucko, Sebastian Kelle, Eckart Fleck, Rolf Gebker

**Affiliations:** 1 Cardiology, German Heart Institute, Berlin, Germany; 2 Cardiology, Hospital of Lithuanian University of Health Sciences, Kaunas, Lithuania; 3 Clinical Science, Philips Helath Care, Hamburg, Germany

## Background

Cardiovascular magnetic resonance (CMR) tissue tagging has been established as non-invasive technique for accurate measurement of myocardial motion. However, additional tagging sequences are necessary and the postprocessing procedure is time consuming. Recently, the novel technique of feature tracking (FT) was introduced. With FT myocardial strain is derived directly from standard steady-state free precession (SSFP) sequences. First studies in children showed a good agreement between tagging and FT for the measurement of circumferential strain (cc). But these results are limited for the circumferential strain of the medial slice.

Aim of this study was to compare more comprehensive tagging data with data derived by FT in adult patients with high grade aortic stenosis (AS).

## Methods

A total number of 28 patients with severe AS underwent cardiac MRI at 1.5 T (Philips Achieva). SSFP images were performed for the short and long axis. Tagging of three short axis planes (apical, medial, basal) was acquired using the CSPAMM technique. The serial short axis slices were used for the assessment of LVEDV, LVESV, LV mass and function.

## Results

Mean LVEF (56±14%), LVEDV (106±58ml) and LVESV (47±48ml) were normal. All patients demonstrated concentric hypertrophy of the LV with an increased basal septal thickness (15.5±2.6mm; LV mass 126±40g/68±23g/m2, papillary muscles excluded).

**Figure 1:**
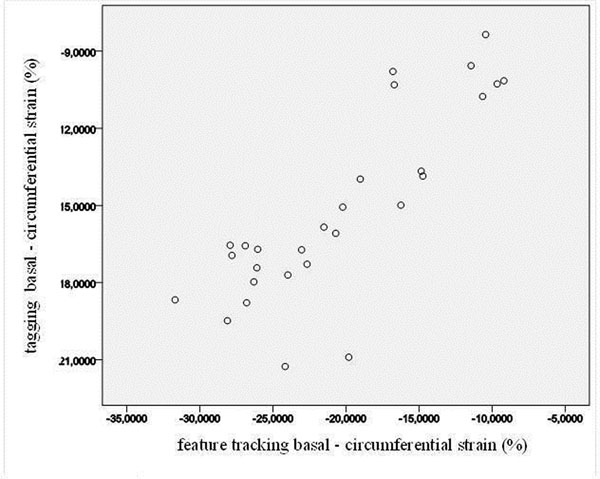


A highly significant correlation was observed between tagging and FT for the derived basal and medial cc (Spearman`s correlation coefficient for basal was 0.81 (figure 1) and for medial 0.742). Apical cc measurements showed a weak correlation (0.35). For different other tagging parameters (rotation, torsion, peak systolic and enddiastolic rotation velocity, time to peak systolic and diastolic rotation velocity, systolic and diastolic strain rate) no correlation was found.

The interobserver agreement for FT showed good and moderate results for cc (kappa for basal 0.6, for medial 0.7 and for apical 0.5) and systolic/early diastolic strain rate (kappa 0.7/0.5).

## Conclusions

To our knowledge is this the first study, which compares different tagging parameters beyond medial cc with FT. FT is a promising method to assess cc using the medial and basal slice in patients with AS. We found a highly significant correlation for the medial cc, which is in agreement with previous published data. Moreover the basal cc showed an excellent correlation, while the correlation for the apical cc was weak. This may be caused by myocardial hypertrophy with less apical endsystolic ventricular volume in patients with AS.

No correlation was observed for different other tagging parameters beyond cc. This could be based on the different technical methods and is hampered by the limited number of patients included.

## Funding

no funding

